# Comparison between two amplicon-based sequencing panels of different scales in the detection of somatic mutations associated with gastric cancer

**DOI:** 10.1186/s12864-016-3166-4

**Published:** 2016-10-26

**Authors:** Yosuke Hirotsu, Yuichiro Kojima, Kenichiro Okimoto, Kenji Amemiya, Hitoshi Mochizuki, Masao Omata

**Affiliations:** 1 Genome Analysis Center, Yamanashi Prefectural Central Hospital, 1-1-1 Fujimi, Kofu, Yamanashi 400-8506 Japan; 2 Department of Gastroenterology, Yamanashi Prefectural Central Hospital, 1-1-1 Fujimi, Kofu, Yamanashi 400-8506 Japan; 3 Department of Gastroenterology and Nephrology, Graduate School of Medicine, Chiba University, 1-8-1 Inohana, Chuo-ku, Chiba 260-8677 Japan; 4 The University of Tokyo, 7-3-1 Hongo, Bunkyo-ku, Tokyo, 113-8655 Japan

**Keywords:** Endoscopic submucosal dissection, Endoscopy, Gastric cancer, Ion PGM, Ion Proton, Mutation, Next-generation sequencing, Targeted sequencing, Tumor

## Abstract

**Background:**

Sequencing data from The Cancer Genome Atlas (TGCA), the International Cancer Genome Consortium and other research institutes have revealed the presence of genetic alterations in several tumor types, including gastric cancer. These data have been combined into a catalog of significantly mutated genes for each cancer type. However, it is unclear to what extent significantly mutated genes need to be examined for detecting genetic alterations in gastric cancer patients. Here, we constructed two custom-made sequencing panels of different scales, the Selective ﻿hotspot﻿ Panel and the Comprehensive Panel, to analyze genetic alterations in 21 resected specimens endoscopically obtained from 20 gastric cancer patients, and we assessed how many mutations were detectable using these different panels.

**Results:**

A total of 21 somatic mutations were identified by the Selective hotspot Panel and 70 mutations were detected by the Comprehensive Panel. All mutations identified by the Selective hotspot Panel were detected by the Comprehensive Panel, with high concordant values of the variant allelic fraction of each mutation (correlation coefficient, *R* = 0.92). At least one mutation was identified in 13 patients (65 %) by the Selective hotspot Panel, whereas the Comprehensive Panel detected mutations in 19 (95 %) patients. Library preparation and sequencing costs were comparable between the two panels.

**Conclusions:**

Our results indicate the utility of comprehensive panel-based targeted sequencing in gastric cancer.

**Electronic supplementary material:**

The online version of this article (doi:10.1186/s12864-016-3166-4) contains supplementary material, which is available to authorized users.

## Background

Gastric cancer is the third- and fifth-highest cause of cancer mortality in men and women, respectively, and accounts for 8 % of total cancer cases and 10 % of total cancer-related deaths worldwide [1]. The highest incidence rates of gastric cancer are in Eastern Asia, Eastern Europe, and South America, while the lowest rates are in North America and most parts of Africa [1]. Major risk factors include *Helicobacter pylori* and Epstein–Barr virus infection, as well as dietary factors such as excessive salt intake [2, 3].

Gastric cancer develops in a step-wise manner, involving chronic gastritis, atrophy, intestinal metaplasia, and dysplasia [4]. Early gastric cancer presents as a malignant tumor confined to the mucosa or submucosa, regardless of the presence of regional lymph node metastasis [5, 6]. The detection of early gastric cancer has recently improved, following the development of endoscopic techniques [7, 8]. In particular, endoscopic submucosal dissection (ESD) has enabled a high *en bloc* resection rate for small and large lesions, as well as in patients with scarring. Moreover, the specimens obtained by ESD can be used for a histological assessment of curability [9]. Endoscopic resection is now widely accepted as a low invasive method for the local resection of early gastric cancer with a negligible risk of lymph node metastasis [10, 11]. Endoscopically-resected early gastric cancer also provides suitable material for genomic analysis to better understand the molecular and genetic features of the initial event leading to cancer development [12].

Next-generation sequencing (NGS) technology enables us to determine the sequence of the genome at a range of different scales, including whole genome, whole exome, and the targeted sequencing of multiple regions of interest. Whereas large-scale analyses are essential for discovery projects, targeted sequencing can focus on genes associated with disease and may lead to advances in the molecular diagnostics of cancer [13]. As an example, NGS has identified a subset of driver and tumor suppressor genes associated with several cancer types [14]. It can also produce thousands to millions of short sequence reads that are massively parallel, and offers a cost-effective approach for detecting genetic alterations.

Large amounts of sequencing data have been disclosed from The Cancer Genome Atlas (TCGA), the International Cancer Genome Consortium (ICGC) and other research institutes. Analyses of these data identified significantly mutated genes (SMGs) in several cancer types [15, 16]. Although SMGs have been revealed by whole exome and whole genome sequencing data, it is unclear to what extent SMGs need to be examined for detecting genetic alterations in gastric cancer. In the present study, we used gastric cancer-associated SMGs to construct two sequencing panels of different scales [17–23]. We performed targeted sequencing and analyzed genetic alterations in gastric tumors at an early phase and assessed how many mutations were detectable using these different panels.

## Methods

### Patients and sample preparation

This study included 20 patients who were diagnosed with gastric cancer (16 males and four females; age 60–87 years) at our hospital (Yamanashi, Japan), one of whom had two tumors. Informed consent was obtained from all subjects. This study was approved by the Institutional Review Board at our hospital and complied with Declaration of Helsinki principles. Peripheral blood samples were obtained from gastric cancer patients and DNA extraction was performed as previously described [24]. Briefly, peripheral blood samples were centrifuged at 820 × *g* at 25 °C for 10 min, and buffy coats were isolated and stored at −80 °C until required for DNA extraction. Buffy coat DNA was extracted using the QIAamp DNA Blood Mini QIAcube Kit (Qiagen, Hilden, Germany) with the QIAcube (Qiagen). The concentration of DNA was determined using the Nano Drop 2000 spectrophotometer (Thermo Fisher Scientific, Waltham, MA).

### Laser capture microdissection and histology

Tumor samples were fixed using 10 % buffered formalin. Serial sections of 10-μm-thick, formalin-fixed, paraffin-embedded (FFPE) tissue were stained with hematoxylin and eosin, and then microdissected using an ArcturusXT laser capture microdissection system (Thermo Fisher Scientific) using ESD-resected specimens. Tumor cells from endoscopic biopsy samples were obtained from 25 serial sections because of the high tumor content. Tumor DNA was extracted using the QIAamp DNA FFPE Tissue Kit (Qiagen).

### DNA quality analysis

The integrity of purified DNA from FFPE samples was assessed using the TaqMan RNase P Detection Reagents kit and the FFPE DNA QC Assay v2 on the ViiA 7 Real-Time PCR System (Thermo Fisher Scientific). Human control genomic DNA included in the TaqMan RNase P Detection Reagents Kit was diluted to create a five-point serial dilution for a standard curve, and absolute DNA concentrations were determined. DNA fragmentation was estimated as the ratio of DNA (relative quantification; RQ) obtained for the long amplicon to the short amplicon. High RQ values indicated that the genomic DNA was intact and high quality.

### Selecting genes and primer design

We searched the literature and selected genes based on the following criteria (Additional file 1: Table S1): (a) SMGs relative to the background mutation rates analyzed by MutSigCV analysis tool [17]; (b) genes involved in signaling pathways and potential therapeutic targets in gastric cancer; and (c) known drivers of gastric carcinogenesis reported by TCGA [17] and other projects [18–22]. We examined the hotspot mutation site of each gene in gastric cancer from the COSMIC database (http://cancer.sanger.ac.uk/cancergenome/projects/cosmic).

We selected 20 genes for the Selective hotspot Panel, which comprises a subset of SMGs and genes related to receptor tyrosine kinases (RTKs) and RAS signaling pathway based on the TCGA project [17]. To expand and cover more SMGs, we selected 58 genes (which include the 20 genes in the Selective hotspot Panel) based on published data from TCGA and another research institute [17–23]. Ion AmpliSeq designer software (Thermo Fisher Scientific) was used to design two custom sequencing panels: the Selective hotspot Panel targeting 20 genes in gastric cancer and the Comprehensive Panel targeting 58 genes [17–23] (Table 1). A total of 376 and 3515 primer pairs were contained within the Selective hotspot Panel (covering 38.01 kb) and the Comprehensive Panel (covering 351.05 kb), respectively.

**Table 1:** Targeted sequencing panels and the analyzed genes associated with gastric cancer

Panel name	Targets size	No. of Amplicons	No. of genes	Covered rate	Gene list
Selective hotspot Panel	38.01 kb	376	20	99.99 %	APC*, ARID1A, BCOR, CDH1*, CTNNB1*, EGFR*, ERBB2*, ERBB3, FGFR2*, JAK2*, KRAS*, MET*, NRAS*, PIK3CA*, PTEN*, RASA1, RHOA*, RNF43, SMAD4*, TP53*
Comprehensive Panel	351.05 kb	3515	58	96.86 %	*ABCA10, ACVR2A, AKAP13,* ***APC*** *, ARHGAP5,* ***ARID1A*** *,* ***BCOR*** *, BNC2, CD274,* ***CDH1*** *, CNGA4, CTNNA1, CTNNA2,* CTNNB1* *, DLC1, DNAH7,* ***EGFR*** *, EIF2C4, ELF3,* ***ERBB2*** *,* ***ERBB3*** *, EYA4, FAM46D, FAT4, FGFR1,* ***FGFR2*** *, GLI3,* ***JAK2*** *, KIF2B, KMT2A, KMT2C,* KRAS* *, LDOC1, MACF1,* ***MET*** *, MUC6,* NRAS* *, PCDH9, PDCD1LG2,* ***PIK3CA*** *, PIK3R1, PKHD1, PLB1,* ***PTEN*** *, PTPRC,* ***RASA1*** *, RGNEF,* RHOA* *,* ***RNF43*** *, SMAD2,* ***SMAD4*** *, SOHLH2, SYNE1, TGFBR2, TMPRSS2,* ***TP53*** *, VEGFA, ZIC4*

Genes shown in bold font represent the 20 identified by the Selective hotspot Panel

*Genes targeting hotspot regions

### Targeted sequencing

Targeted sequencing was performed as previously described [25]. Multiplex polymerase chain reaction (PCR) of these panels was performed using the Ion AmpliSeq Library Kit 2.0 (Thermo Fisher Scientific). Primer sequences were digested with FuPa reagent (Thermo Fisher Scientific), and then barcoded using Ion Xpress Barcode Adapters (Thermo Fisher Scientific). Purification was carried out by Agencourt AMPure XP reagents (Beckman Coulter, Brea, CA). The library concentration was determined using an Ion Library Quantitation Kit (Thermo Fisher Scientific); each library was diluted to 10 pM, and the same amount of libraries was pooled for one sequence reaction. Emulsion PCR was carried out using the Ion OneTouch System and Ion PGM Template OT2 200 kit or Ion PI Template OT2 200 Kit v3 (Thermo Fisher Scientific). Template-positive Ion Sphere Particles were then enriched using the Ion OneTouch ES system (Thermo Fisher Scientific), and purified Ion Sphere particles were loaded on an Ion 318 Chip v2 or PI Chip (Thermo Fisher Scientific). Massively parallel sequencing was carried out on Ion PGM or Ion Proton systems (Thermo Fisher Scientific).

### Data analysis

Sequence data were processed using standard Ion Torrent Suite Software running on the Torrent Server. Raw signal data were analyzed using Torrent Suite version 4.4. The data processing pipeline involved signaling processing, base calling, quality score assignment, adapter trimming, PCR duplicate removal, read alignment to the human genome 19 reference (hg19), quality control of mapping quality, coverage analysis, and variant calling. Following data analysis, the annotation of single nucleotide variants, insertions, and deletions was performed by the Ion Reporter Server System (Thermo Fisher Scientific), and peripheral blood DNA was used as a control to detect variants in tumors (Tumor–Normal pairs). We used the following filtering parameters for variant calling: the minimum number of variant allele reads was ≥5, the coverage depth was ≥10, and the variant allele fraction was ≥10 %. If somatic mutations were called using either the Selective hotspot Panel or Comprehensive Panel, sequence data were visually confirmed with the Integrative Genomics Viewer and any sequence, alignment, or variant call error artifacts were discarded.

## Results

### Quality assessment of extracted FFPE DNA

**Fig. 1:**
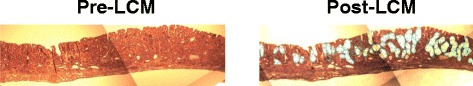
Representative image of microdissected specimen. Tumor cells were obtained from ESD-resected specimens using laser capture microdissection (LCM). Left image (Pre-LCM) is before microdissection; right image is after microdissection (Post-LCM). Cyan circles indicate the cutting area

We examined 21 FFPE tumor samples collected from 20 patients (early stage, 19 patients; advanced stage, one patient) who had not previously undergone chemotherapy or radiotherapy. Matched peripheral blood lymphocytes were included as a control. Of the 21 FFPE tumor samples, 19 tumors had been resected by ESD and two by endoscopic biopsy. ESD-resected tumor tissue was dissected by laser capture microdissection with an average cutting area of 29.4 mm^2^ (range, 12.4–51.5 mm^2^) (Fig. 1 and Additional file 1: Table S2). Endoscopic biopsy samples were not microdissected because of the high tumor content.

To assess the extent of DNA degradation, we performed quantitative real-time PCR using two primer pairs (short amplicon, 87 bp; long amplicon, 268 bp) flanking the human RNase P locus [26, 27]. Short and long DNA fragment yields were estimated as 14.4 ng/μL (range, 0.6–65.0 ng/μL) and 8.0 ng/μL (range, 0.2–35.8 ng/μL), respectively (Additional file 1: Table S3). An estimate of FFPE-derived genomic DNA fragmentation using the RQ gave an average value of 0.49 (range, 0.14–0.73) (Additional file 1: Table S3), indicating that DNA of high quality had been extracted from FFPE specimens.

### Targeted sequencing analysis

To identify genetic alternations in gastric cancer, we reviewed cancer genome sequences from TCGA, ICGC, and COSMIC databases, and selected all SMGs associated with gastric cancer. We constructed two custom-made gastric cancer panels. The Selective hotspot Panel spans 38,010 nucleotides, covers 20 SMGs, and mainly targets hotspot regions (Table 1). The Comprehensive Panel spans 354,050 nucleotides, and 58 of the genes contained within this panel overlapped with the Selective hotspot Panel (Table 1).

We performed targeted sequencing using the two panels with a next-generation sequencer (Ion Proton or Ion PGM, Thermo Fisher Scientific). The percentage of mapped reads aligned to target regions was 98.7 % (97.6–99.3 %) in the Selective hotspot Panel and 97.0 % (95.0–98.6 %) in the Comprehensive Panel, suggesting that all FFPE-derived DNAs had been successfully subjected to library preparation following sequencing analysis (Table 2).

**Table 2:** Coverage depth of the data from the two panels

		Selective hotspot Panel				Comprehensive Cancer Panel			
Case	Sample	Mapped Reads	On Target	Mean Depth	Uniformity	Mapped Reads	On Target	Mean Depth	Uniformity
Case 1	Buffy coat	484124	98.0 %	1402	94.0 %	1097404	96.4 %	307	96.0 %
Case 2	Buffy coat	426768	98.1 %	1233	94.0 %	1267404	96.3 %	355	96.1 %
Case 3	Buffy coat	518868	98.2 %	1507	93.8 %	810553	96.3 %	221	95.0 %
Case 4	Buffy coat	542769	98.4 %	1576	93.9 %	379011	96.1 %	106	94.5 %
Case 5	Buffy coat	624920	97.9 %	1796	94.3 %	11170406	96.8 %	3112	95.1 %
Case 6	Buffy coat	164634	98.9 %	477	93.5 %	2069779	97.9 %	611	90.6 %
Case 7	Buffy coat	126183	99.0 %	363	91.0 %	2256973	97.8 %	666	91.0 %
Case 8	Buffy coat	108641	99.1 %	315	90.5 %	2027465	97.7 %	601	92.0 %
Case 9	Buffy coat	166684	99.0 %	487	94.7 %	1857285	97.8 %	551	92.2 %
Case 10	Buffy coat	107898	99.0 %	314	90.7 %	2013379	98.0 %	593	89.0 %
Case 11	Buffy coat	79551	99.2 %	231	87.1 %	2106246	98.1 %	624	88.7 %
Case 12	Buffy coat	67486	99.2 %	197	88.3 %	1859173	98.3 %	542	82.5 %
Case 13	Buffy coat	60485	99.2 %	175	91.2 %	1918204	98.3 %	548	78.6 %
Case 14	Buffy coat	260250	98.8 %	759	92.4 %	1295372	96.7 %	360	94.9 %
Case 15	Buffy coat	235410	98.7 %	680	94.2 %	1053705	96.9 %	293	94.6 %
Case 16	Buffy coat	246227	99.0 %	715	93.7 %	845571	97.0 %	234	94.7 %
Case 17	Buffy coat	268465	98.8 %	779	94.4 %	1223358	96.8 %	341	95.2 %
Case 18	Buffy coat	280097	99.1 %	811	93.9 %	3130126	97.0 %	902	93.6 %
Case 19	Buffy coat	256281	98.9 %	744	94.5 %	3423132	97.0 %	987	94.4 %
Case 20	Buffy coat	207402	98.9 %	598	92.4 %	2913580	97.0 %	830	93.6 %
	Mean ± SD	261657 ± 170658	98.7 ± 0.42 %	758 ± 493	92.6 ± 2.1 %	2235906 ± 2247475	97.2 ± 0.71 %	639 ± 627	92.1 ± 4.6 %
Case 1	Tumor	403252	98.5 %	1143	87.8 %	2025838	97.7 %	522	71.0 %
Case 2	Tumor	426999	98.4 %	1222	91.6 %	999656	96.9 %	276	94.1 %
Case 3	Tumor	524706	98.5 %	1502	91.8 %	1032722	96.3 %	281	94.6 %
Case 4	Tumor	467625	98.0 %	1337	91.0 %	789820	95.8 %	210	95.2 %
Case 5	Tumor	412941	98.5 %	1186	90.7 %	829933	96.0 %	223	94.9 %
Case 6	Tumor	72752	99.3 %	207	84.8 %	1949114	98.6 %	577	83.2 %
Case 7	Tumor	119809	99.3 %	340	85.8 %	2511041	97.6 %	718	91.2 %
Case 8	Tumor	85768	99.2 %	246	88.2 %	1521154	98.6 %	437	82.4 %
Case 9	Tumor	182739	99.2 %	523	89.0 %	2048473	98.2 %	583	88.3 %
Case 10	Tumor	110281	98.0 %	311	88.5 %	2702014	97.3 %	762	87.2 %
Case 11	Tumor	96338	99.2 %	273	89.8 %	2668972	98.0 %	750	86.1 %
Case 12	Tumor	106265	99.2 %	303	87.7 %	2382665	97.8 %	673	89.0 %
Case 13	Tumor site1	87099	98.8 %	250	92.4 %	2861114	97.5 %	819	91.1 %
Case 13	Tumor site2	138279	99.3 %	395	88.9 %	2705723	97.9 %	777	91.5 %
Case 14	Tumor	230616	98.9 %	662	92.6 %	781688	96.5 %	211	95.1 %
Case 15	Tumor	262428	99.1 %	742	65.8 %	631147	96.6 %	171	95.2 %
Case 16	Tumor	179627	99.1 %	512	87.8 %	3189103	95.9 %	929	95.8 %
Case 17	Tumor	153577	99.0 %	434	85.7 %	539462	96.5 %	145	95.0 %
Case 18	Tumor	145284	98.4 %	411	94.7 %	2514172	96.4 %	697	91.0 %
Case 19	Tumor	184785	97.6 %	520	94.0 %	2333968	95.0 %	637	91.4 %
Case 20	Tumor	105210	97.8 %	294	95.0 %	1663278	96.6 %	436	80.3 %
	Mean ± SD	214113 ± 143314	98.7 ± 0.54 %	610 ± 411	88.7 ± 6.0 %	1841955 ± 850360	97.0 ± 1.0 %	516 ± 247	89.7 ± 6.3 %

**Fig. 2:**
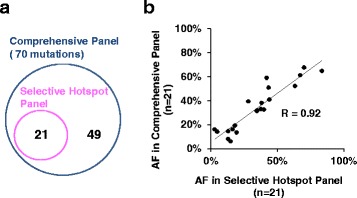
Correlation of variant allele fractions detected in the two panels. Panel **a**: Venn diagram of identified mutations in the two panels. Twenty-one variants identified by the Selective hotspot Panel were also detected by the Comprehensive Panel. Panel **b**: Comparison of variant allelic fractions (AF) between the two panels. The AF value of 21 variants is plotted. The correlation coefficient (R) is 0.92

The mean coverage depth of tumors was 610× (range, 207–1502) by the Selective hotspot Panel, and 516× (range, 145–923) by the Comprehensive Panel (Table 2). The two approaches identified a total of 21 and 70 somatic mutations in tumors, respectively (Fig. 2a and Table 3). All 21 mutations identified by the Selective hotspot Panel were also confirmed by the Comprehensive Panel (Fig. 2a). The variant allelic fraction values were significantly correlated between the two panels (Fig. 2b). Seventy mutations were detected in the 21 tumors. Overall, an average of 3.2 mutations (range, 0–8) were detected in each early gastric tumor, whereas seven mutations were detected in the advanced tumor. At least one mutation was detected in 13 of the 20 patients (65 %) by the Selective hotspot Panel, and in 19 of the 20 patients (95 %) by the Comprehensive Panel. These results suggest that the Comprehensive Panel covered the genetic alterations of almost all gastric cancer patients.

**Table 3:** Somatic mutations identified using the two panels

Case	Specimen	Characteristics	Gene	Mutation	Selective hotspot panel, allelic fraction	Comprehensive panel, allelic fraction
Case 1	ESD	Early	*MUC6*	F1843S	Not included	28 %
			*MUC6*	S1531P	Not included	23 %
			*PKHD1*	F83S	Not included	11 %
Case 2	ESD	Early	*ZIC4*	R107H	Not included	21 %
			*RASA1*	K825X	Not included	21 %
Case 3	ESD	Early	*MACF1*	L2900F	Not included	11 %
Case 4	ESD	Early	*APC*	C207X	Not included	34 %
			*APC*	Q1447X	35 %	31 %
			*MUC6*	A1637Q	Not included	16 %
			*SYNE1*	D5070G	Not included	13 %
			*PKHD1*	Q3467K	Not included	12 %
			*MUC6*	L1836H	Not included	10 %
Case 5	ESD	Early	*SYNE1*	G474R	Not included	83 %
			*TP53*	G266V	83 %	65 %
			*PKHD1*	R723C	Not included	32 %
			*FAM46D*	S69C	Not included	16 %
Case 6	ESD	Early	*MUC6*	T2041M	Not included	33 %
			*SMAD2*	A278P	Not included	17 %
			*MACF1*	R3680K	Not included	14 %
Case 7	ESD	Early	*TP53*	D148fs	67 %	61 %
Case 8	ESD	Early	*APC*	L1564X	42 %	59 %
			*SYNE1*	D903Y	Not included	43 %
			*APC*	S940X	Not included	42 %
			*TMPRSS2*	L141V	Not included	40 %
			*AKAP13*	A2256V	Not included	28 %
			*MUC6*	T2041M	Not included	25 %
			*MUC6*	P1571T	Not included	21 %
Case 9	ESD	Early	*APC*	Q1237fs	Not included	38 %
Case 10	ESD	Early	*TP53*	H193Y	63 %	52 %
			*APC*	S1068X	Not included	42 %
			*SMAD4*	G477X	Not included	35 %
			*KRAS*	G13D	38 %	33 %
			*RHOA*	M1V	13 %	14 %
			*APC*	Q1517fs	5 %	14 %
Case 11	ESD	Early	*ELF3*	D220N	Not included	42 %
			*SYNE1*	K874N	Not included	40 %
			*SMAD4*	R497H	28 %	39 %
			*FAT4*	K225E	Not included	35 %
			*KMT2C*	Y987H	Not included	33 %
			*ERBB2*	R897Q	Not included	32 %
			*SYNE1*	R7753H	Not included	32 %
			*MET*	N381fs	19 %	13 %
Case 12	ESD	Early	*APC*	R876X	38 %	38 %
			*MUC6*	T2041M	Not included	28 %
Case 13_site1^a^	ESD	Early	*TP53*	R209fs	70 %	67 %
			*ARHGAP5*	L297X	Not included	31 %
			*PKHD1*	I3786M	Not included	12 %
Case 13_site2^a^	ESD	Early	*TP53*	E258D	Not included	71 %
			*MACF1*	D968A	Not included	51 %
Case 14	ESD	Early	-	-	Not detected	Not detected
Case 15	ESD	Early	*SYNE1*	R6836C	Not included	51 %
			*ACVR2A*	R202fs	Not included	39 %
			*MUC6*	S2378fs	Not included	38 %
			*DLC1*	E854K	Not included	17 %
Case 16	ESD	Early	*ARID1A*	K1072fs	43 %	51 %
			*RASA1*	R512X	44 %	41 %
			TP53	G154S	35 %	32 %
			*RASA1*	D380E	18 %	19 %
			*MUC6*	P1724S	Not included	15 %
			*ARHGAP5*	L259S	Not included	10 %
Case 17	ESD	Early	*CTNNB1*	S45F	40 %	32 %
Case 18	ESD	Early	*TP53*	N200fs	3 %	16 %
Case 19	Biopsy	Early	*TP53*	R175H	15 %	6 %
			*APC*	E262X	Not included	12 %
Case 20	Biopsy	Advanced	*TGFBR2*	S94R	Not included	28 %
			*CDH1*	Splice site	Not included	28 %
			*MACF1*	G5253E	Not included	19 %
			*TP53*	R248Q	17 %	16 %
			*DNAH7*	Y2563N	Not included	13 %
			*DLC1*	W10L	Not included	13 %
			CDH1	Splice site (c.1009-2A>C)	13 %	8 %

^a^Case 13 had two tumors

### Running costs

Primer costs for the Comprehensive Panel were higher than those of the Selective hotspot Panel (Comprehensive Panel: $26363 vs. Selective hotspot Panel: $2820). However, the total cost of library preparation, emersion PCR, and massively parallel sequencing was comparable between the two panels at $200–250 per sample. Use of the Barcode Xpress toolkit enabled multiple samples to be simultaneously sequenced in 4–5 h and allowed us to obtain high-depth sequence data using the Ion PGM or Ion Proton system.

## Discussion

The identification of oncogenic driver genes has led to the development of potent molecular targeting drugs together with companion diagnostics. The advent of NGS has also resulted in the identification of a subset of cancer-related genes in several tumors [14, 15], including hundreds of genes mainly associated with tumor development [28]. TCGA, ICGC, and other research institutes have revealed a tumor mutational landscape and produced a catalog of somatic mutations associated with tumors. Information from this catalog has enabled the analysis of recurrently mutated genes by targeted sequencing [29]. This is a useful, cost-effective method for identifying variants in dozens to hundreds of genes, and is fairly readily available for routine diagnosis in a clinical setting as well as for research purposes.

In this study, we constructed two amplicon-based targeted panels of different scales to analyze the genetic alterations associated with gastric cancer. In our cohort, 20 out of 21 tumors (95 %) were shown to carry at least one mutation by the Comprehensive Panel. Thus, our panel-based approach enabled us to detect somatic mutations in gastric cancer, suggesting that it has the potential to obtain robust data and to detect genetic events in tumors. Furthermore, two patients (10 %) harbored mutations in potential therapeutic targets such as *KRAS* (5 %), *ERBB2* (5 %) and *MET* (5 %) [17, 23]. With the increasing numbers of molecular targeting drugs under development or clinical trial, Comprehensive Panels may offer better selection for molecular-targeted therapy for gastric cancer patients. Collectively, this demonstrates the utility of targeted sequencing using a multi-gene panel in cancer genome research and clinical settings.

Progress in endoscopic technology has led to the curative resection of gastric cancer at an early stage. However, although ESD is widely performed to resect early gastric cancer, the genetic alterations occurring in such tumors are not fully understood, even though this would provide us with an insight into the mechanisms of tumorigenesis. Here, we performed targeted sequencing using ESD-resected early gastric cancers, together with endoscopically-resected biopsies of advanced cancer. A total of 70 somatic mutations were identified in 19 patients, and an average 3.2 mutations were found in early gastric cancer. The most recurrent mutation was identified in *TP53* gene (43 %, 9/21). In line with this observation, previous studies have shown that *TP53* mutations occur in early gastric cancer as well as in high-grade intraepithelial neoplasia [30]. These observations indicate that *TP53* is a key molecule for the progression of gastric tumorigenesis.

In this study, somatic mutations in *TP53* (43 %), *APC* (29 %), *MUC6* (33 %), and *SYNE1* (24 %) were frequently observed (identified in over 20 % of tumors). These frequencies are almost consistent with previous studies that reported mutations in *TP53* (36–73 %), *APC* (5–14 %), *MUC6* (6–18 %), and *SYNE1* (20 %). Less common mutations were observed in *CTNNB1* (5 %) and *KRAS* (5 %) genes in our study, but these gene mutations (*CTNNB1* S45F and *KRAS* G13D) are well-known hotspot driver mutations [31]. Previous data also showed that *CTNNB1* (1–9 %) and *KRAS* (5–6 %) mutations were relatively uncommon in gastric cancer. These results indicated that our designed panels validated the data of previous reports.

The TCGA project demonstrated there are four major subtypes of gastric cancer based on the genomic analysis, i.e., chromosomal instability (CIN), genomically stable (GS), Epstein-Barr virus-positive and microsatellite instability [17]. According to this molecular classification, *TP53* mutation mostly occurs in the CIN category and intestinal histology. Consistent with this, we examined ESD-resected gastric tumors and most were intestinal type gastric cancer (data not shown). Additionally, the GS subtype is classified as diffuse histology and frequently shows *CDH1* and *RHOA* mutations and *CLDN18-ARHGAP* fusion. Again, in our series, one advanced gastric cancer was diffused type histology and had a *CDH1* splice site mutation (Case 20 in Table 3). Collectively, our data reinforced the molecular classifications of gastric cancer.

Analyses that include a large number of SMGs are important for several reasons. First, analyzing additional SMGs will detect more somatic alterations in tumors. In this study, we were unable to identify any mutations in seven patients using the Selective hotspot Panel, compared with only one using the Comprehensive Panel (Table 3). A recent study reported newly identified SMGs including *NRG1*, *ERBB4*, *XIRP2*, *NBEA*, *COL14A1*, *CNBD1*, *ITGAV*, and *AKAP6* [32, 33] that should be included in the mutational spectrum analyzed in all patients with gastric cancer. Second, from a cost perspective, covering more SMGs is beneficial, as shown by the comparable library preparation and sequencing running costs between the two panels used in this study. Third, including more primer pairs in the design of the panel enables more high-resolution copy number data to be examined [34]. Previous bioinformatics analysis combined with variant allelic fraction and copy number alteration data revealed the cellular prevalence of tumor heterogeneity [35]. Together, these findings suggest that SMG-based sequencing analysis is a useful method for further investigating tumor heterogeneity in clinical samples.

## Conclusions

In the present study, use of the Comprehensive Panel covering SMGs associated with gastric cancer enabled the analysis of genetic alterations in patients with early gastric cancer.

## Additional file

Additional file 1: Table S1. Panel description and references. **Table S2.** Cutting areas of ESD-resected specimens (*n*=19) by laser capture microdissection and biopsies (*n*=2). **Table S3.** Assessment of tumor-derived DNA qualities. (XLS 43 kb)

## Abbreviations

ESD: Endoscopic submucosal dissection
FFPE: Formalin-fixed, paraffin-embedded
ICGC: International Cancer Genome Consortium
NGS: Next-generation sequencing
SMGs: Significantly mutated genes
TCGA: The Cancer Genome Atlas
